# Response to Letter to Editor “Critical Response to the Comments on the Critical Issue of Infant and Child Mortality in Afghanistan”

**DOI:** 10.1002/hsr2.71288

**Published:** 2025-09-29

**Authors:** Salvatore Chirumbolo

**Affiliations:** ^1^ Department of Engineering for Innovation Medicine University of Verona Verona Italy

To the Editor,

1

1.1

I appreciate the thoughtful engagement by Giuseppe Lippi with my recent critical analysis regarding the review by Qamar et al. on infant and child mortality in Afghanistan [1], yet, I respectfully disagree with several points raised, and I would like to clarify my perspective and address certain misconceptions presented in their letter.

First, regarding the reliance on secondary data: while I fully acknowledge that primary data collection is extremely challenging in conflict‐affected settings such as Afghanistan, my critique was not directed at the necessity of using secondary sources per se, but rather at the uncritical reliance on them without adequate contextual validation. High‐quality scoping reviews, even when based on secondary data, should rigorously assess the reliability, scope, and potential biases of the sources cited. Simply accepting secondary data at face value risks perpetuating inaccuracies, especially in regions with political instability where data can be incomplete or politicized.

Second, while Lippi et al. emphasize that a scoping review does not require statistical analysis or multivariate evaluation, I respectfully point out that the best practice guidelines for scoping reviews (e.g., PRISMA‐ScR) encourage at least a basic synthesis of quantitative patterns when feasible. My suggestion to explore multifactorial correlations or success stories was not intended to impose unwarranted methodological burdens, but rather to propose a richer, more nuanced interpretation of the existing data, which would have strengthened the review's contribution.

Third, regarding political bias: my commentary regarding the depiction of the Taliban's impact on healthcare infrastructure was not an accusation of partisanship but a call for balanced analysis. Given the complexity of Afghanistan's decades‐long crises, a comprehensive assessment should consider multiple factors (e.g., international sanctions, economic collapse, historical healthcare deficiencies) beyond political blame attribution. The call for greater analytical neutrality was made in the spirit of scientific rigor, not political partiality.

Fourth, it is unsubstantiated to imply that geographic or national origin limits one's ability to assess international public health issues critically. Scientific critique should not be contingent upon one's physical proximity to the country under study, but rather on the quality of the evidence and reasoning presented. The argument that commentary must be restricted to those with “firsthand experience” is neither consistent with international norms of academic discourse nor conducive to the advancement of global health scholarship.

Lastly, my suggestion to improve healthcare infrastructure, including rural facility development, is rooted in long‐standing global health recommendations, not in naïve optimism. While recognizing Afghanistan's dire economic challenges, highlighting future needs is not impractical—it is essential for comprehensive policy planning and advocacy, even in resource‐limited settings.

In conclusion, my intent was, and remains, to encourage deeper critical engagement with a vital topic, respecting the difficult context of Afghanistan while upholding rigorous scientific standards. I remain committed to constructive dialog aimed at improving the quality and impact of health research for vulnerable populations worldwide. Obviously, as this Reply pertains to a scientific point of view, the same should be considered within the democratic arena of the critical debate in science.

## Author Contributions

**Salvatore Chirumbolo:** conceptualization, investigation, writing – original draft, methodology, validation, visualization, writing – review and editing, formal analysis, software, supervision.

## Conflicts of Interest

The author declares no conflicts of interest.

## Transparency Statement

Salvatore Chirumbolo affirms that this manuscript is an honest, accurate, and transparent account of the study being reported; that no important aspects of the study have been omitted; and that any discrepancies from the study as planned (and, if relevant, registered) have been explained.

## Data Availability

Data sharing is not applicable to this article as no data sets were generated or analyzed during the current study. Salvatore Chirumbolo (Corresponding author) had full access to all of the data in this study and takes complete responsibility for the integrity of the data and the accuracy of the data analysis.

## References

[1] S. Chirumbolo , “Infant and Child Mortality in Afghanistan Wheat and Chaff,” Health Science Reports 8, no. 1 (January 2025): e70382, 10.1002/hsr2.70382. 39846039 PMC11752134

